# The role of bone marrow adipocytes in bone metastasis

**DOI:** 10.1016/j.jbo.2016.03.006

**Published:** 2016-04-08

**Authors:** Emma V. Morris, Claire M. Edwards

**Affiliations:** a Nuffield Dept. of Surgical Sciences, University of Oxford, Oxford, UK; b Nuffield Dept. of Orthopaedics, Rheumatology and Musculoskeletal Sciences, University of Oxford, Oxford, UK

## Abstract

Adipocytes are a significant component of the bone marrow microenvironment. Although bone marrow adipocytes were first identified more than 100 years ago, it is only in recent years that an understanding of their complex physiological role is emerging. Bone marrow adipocytes act as local regulators of skeletal biology and homeostasis, with recent studies suggesting that marrow adipose tissue is metabolically active, and can function as an endocrine organ. As such, bone marrow adipocytes have the potential to interact with tumour cells, influencing both tumour growth and bone disease. This review discusses the current evidence for the role of bone marrow adipocytes in tumour growth within the bone marrow microenvironment and the development of the associated bone disease.

## Highlights

• Bone marrow adipocytes are a metabolically active source of lipids and adipokines.
• Marrow adipocytes increase with age, but their role in bone metastasis is ill-defined.
• Marrow adipocytes have tumour-promoting and -suppressive effects in bone metastasis.

## 1 Introduction

Bone marrow adipocytes are one of the most abundant cell types found in bone marrow tissue. They constitute approximately 15% of the bone marrow volume in young adults, rising to 60% by the age of 65 years old [1]. Previously considered as inert space filling cells with little biological significance, accumulating evidence demonstrates that bone marrow adipocytes are more than just passive bystanders of the marrow. They have a distinctive phenotype, which resembles both brown and white adipose tissue and are now recognised to have specialised functions [2]. They store and secrete fatty acids, cytokines and adipokines among them leptin and adiponectin, which regulate calorie intake and insulin sensitivity, respectively. Morphologically bone marrow adipocytes are smaller in size than their visceral counterparts; however the net effect of fatty acid uptake is similar due to enhanced triacylglycerol synthesis. They have the potential to influence neighbouring cells by autocrine, paracrine and endocrine signalling making them a powerful player in influencing the bone microenvironment as a whole. Marrow adipocytes and osteoblasts share common progenitor cells, known as bone marrow mesenchymal stromal cells (MSCs) [3]. Their lineage commitment is thought to be regulated by adipogenic and osteogenic factors in the bone microenvironment that activate their respective transcriptional programs. However, in recent years the identification of MSC subpopulations that are thought to be lineage committed has added another level of complexity. The balance between these two cell types appears to play a pivotal role in bone homeostasis and so when the scales are tipped in favour of adipogenesis then by default osteoblastogenesis is negatively regulated. Moreover, there is a building body of evidence to suggest that a subpopulation of adipocytes are generated from bone marrow myeloid cells, posing the question as to how these differ in their function and behaviour to adipocytes generated from MSCs [4]. Furthermore, bone marrow adiposity is also known to inhibit haematopoiesis [5]. There is considerable evidence to support a metabolic role for marrow adipocytes however their influence on the development and progression of metastatic bone disease is only now becoming apparent.

## 2 Bone marrow adipocytes in the tumour-bone microenvironment

**Fig. 1:**
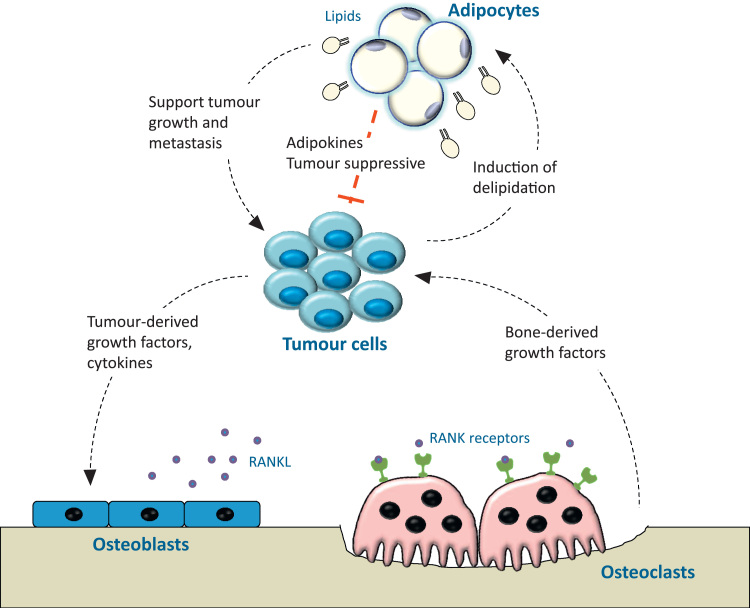
Adipocytes within the tumour-bone microenvironment. The relationship between tumour cells and bone cells is well documented, whereby tumour cells promote osteolytic bone disease and resorbed bone releases factors to promote tumour growth and survival. Adipocytes are ideally placed to interact with this relationship, with evidence to support both pro- and anti-tumour effects, and feedback from tumour cells to bone marrow adipocytes.

The bone provides a unique and supportive microenvironment for a number of solid tumour metastases including breast, prostate and the haematological malignancy multiple myeloma [6]. Cancer cells that intrude into this microenvironment produce various cytokines and growth factors which dysregulate the normal coupling of osteoclasts and osteoblasts. The increased bone resorption releases a number of factors which act positively upon the cancer cells thus perpetuating a “vicious cycle”, a feed-forward cycle that is critical to the establishment of bone metastasis. However, it would be short sighted to think that bone metastases only impinge upon osteoblasts and osteoclasts, as there are many more cells residing in the bone marrow such as fibroblasts, macrophages and adipocytes (Fig. 1) whose contribution should not be ignored. Bone metastatic cancers primarily occur in older patients whose bone marrow is heavily populated by adipocytes [7]. In recent years there has been building interest in the contribution of marrow adipocytes to metastatic disease. In breast, multiple myeloma (MM) and prostate there is demonstrable evidence that marrow adipocytes attract and interact with cancer cells, however the advantages these interactions bestow are still open to debate. Diet-induced obesity has been shown to promote development of a myeloma-like condition, and to increase prostate cancer-induced bone disease [8], [9], [10]. Cancer cells are attracted to adipocytes within the metabolically active red marrow of the bone and these adipocytes interact closely with their neighbouring cells [9], [11], [12], [13]. These observations suggest that an adipocyte-rich environment could fuel disease by creating a permissive favourable niche for cancer cells to establish and progress.

Both breast cancer and MM cause osteolytic lesions, inhibiting osteoblast differentiation and thereby tipping the balance in favour of osteoclastic activity. In contrast, prostate cancer predominantly causes osteoblastic bone disease. Interestingly, increased marrow adiposity has been associated with both osteolytic and osteoblastic disease [8], [10], [14]. However, one crucial factor these two processes have in common is the need for energy. Adipocytes are filled with numerous lipid droplets which serve as an effective source of fatty acids when metabolic demand is increased. Podgorski and colleagues demonstrated that lipids can be trafficked between adipocytes and cancer cells fuelling tumour growth and invasiveness by upregulating FABP4, IL-1β and HMOX-1 in the metastatic tumour cells [9]. Adipocytes also support cancer cells in an endocrine manner, secreting growth factors, adipokines and chemokines that lead to tumour survival. In MM factors such as IL-6, TNF-α, CXCL12 and leptin play a role in disease establishment and progression promoting cell proliferation and migration as well as preventing apoptosis [11], [15]. In prostate cancer the chemokines CXCL1 and CXCL2 have been implicated in promoting tumour associated bone disease by upregulating osteoclastogenesis, and in turn promoting tumour cell survival [10]. Recently, breast cancer cells have been shown to be recruited to bone marrow adipose tissue by the secretion of IL-1β and leptin [16]. The abundance of marrow adipocytes in ageing bones may increase the fertility of the bone microenvironment by providing a constant source of energy and growth factors for cancer cells to thrive and progress in these skeletal sites. However, the identification of bone marrow adipocytes as a major source of circulating adiponectin [17], greater than white adipose tissue, raises the possibility that bone marrow adipocytes may also have anti-tumour functions due to the tumour-suppressive effects of adiponectin.

## 3 Cancer-associated adipocytes

Adipocytes located in close proximity to invasive cancer cells in the primary tumour exhibit profound phenotypic changes that include both morphological and functional alternations and are often referred to as cancer-associated adipocytes (CAAs). The morphological changes associated with these cells include loss of lipid content (delipidation) and acquisition of a fibroblast-like/preadipocyte phenotype (de-differentiation). Functionally they exhibit a decrease in expression of adipocyte-related genes such as adiponectin, FABP4 and resistin coupled with an increase in the production of pro-inflammatory cytokines IL-6, IL-1β [9], [18]. These changes were primarily reported in breast cancer studies in associated with white adipose tissue, however recent *in vitro* work suggests these changes are also important in the bone marrow [9].

## 4 Targeting adipocytes

Given the potential tumour-supporting role of adipocytes, targeting these cells either alone or in combination with common therapeutics may be a promising approach. Modulating levels of adipokines such as adiponectin has been shown to exert an anti-tumour effect. Pharmacological enhancement of circulating adiponectin by the apolipoprotein mimetic L-4F was shown to cause cancer cell death in mouse models of myeloma [19]. Due to the increasing importance of lipid metabolism in tumour cell survival, drugs have been developed that target essential molecules of fatty acid synthesis and uptake. Chemical or RNAi-mediated inhibition of key enzymes involved in fatty acid synthesis, including fatty acid synthase (FASN) [20], acetyl-CoA-carboxylase and ATP-citrate lyase has been shown to attenuate tumour cell proliferation and induce cell death in a number of different cancer cell lines and mouse models [21]. Approaches that regulate the balance between adipogenesis and osteogenesis may also be effective in maintaining healthy bone homeostasis thereby preventing cancer infiltration. Modulation of the nuclear receptors, glucocorticoid receptor and PPARγ and their respective pharmacological ligands, corticosteroids and thiazolidinediones, directly regulate osteogenic versus adipogenic differentiation of MSCs [22] and so could be targeted accordingly. Another such target is protein kinase C which also promotes osteogenesis and has anti-tumourigenic properties [23]. Investigating these treatment strategies more closely may provide new insight in to which pathways are being exploited by cancer cells in order to evade conventional treatments. Targeting adipocytes as part of a combination therapy may prove to be a valuable tool, however a greater understanding between the balance of tumour-promoting and tumour-suppressive effects of bone marrow adipocytes is required.

## 5 Conclusions

Over the last few decades the contribution of adipocytes to disease establishment and progression has become clearer. With aging and obesity resulting in increased numbers of bone marrow adipocytes, it is important to further understand the influence these cells are having on their environment. Targeting adipocytes and their products may open new therapeutic avenues in the fight against lethal metastatic cancers.
